# Attentional Guidance from Multiple Working Memory Representations: Evidence from Eye Movements

**DOI:** 10.1038/s41598-018-32144-4

**Published:** 2018-09-17

**Authors:** Bao Zhang, Shuhui Liu, Mattia Doro, Giovanni Galfano

**Affiliations:** 1 Department of Psychology and the Center for Mind and Brain, Guangzhou University, Guangzhou, China; 2 Department of Developmental and Social Psychology, University of Padova, Padova, Italy

## Abstract

Recent studies have shown that the representation of an item in visual working memory (VWM) can bias the deployment of attention to stimuli in the visual scene possessing the same features. When multiple item representations are simultaneously held in VWM, whether these representations, especially those held in a non-prioritized or accessory status, are able to bias attention, is still controversial. In the present study we adopted an eye tracking technique to shed light on this issue. In particular, we implemented a manipulation aimed at prioritizing one of the VWM representation to an active status, and tested whether attention could be guided by both the prioritized and the accessory representations when they reappeared as distractors in a visual search task. Notably, in Experiment 1, an analysis of first fixation proportion (FFP) revealed that both the prioritized and the accessory representations were able to capture attention suggesting a significant attentional guidance effect. However, such effect was not present in manual response times (RT). Most critically, in Experiment 2, we used a more robust experimental design controlling for different factors that might have played a role in shaping these findings. The results showed evidence for attentional guidance from the accessory representation in both manual RTs and FFPs. Interestingly, FFPs showed a stronger attentional bias for the prioritized representation than for the accessory representation across experiments. The overall findings suggest that multiple VWM representations, even the accessory representation, can simultaneously interact with visual attention.

## Introduction

The notion that visual working memory (VWM) plays a critical role in visual search is well established and strongly supported by empirical evidence (see the review by Woodman & Chun^1^). VWM is generally assumed to maintain a target template during visual search, and allows the comparison between the attended items and the target representation^2,3^. As a result, the template in VWM will guide attention to the items in the visual scene that are more similar to the target, hence promoting visual search efficiency. In addition, items maintained in VWM and irrelevant to the search target, have also been shown to bias attentional deployment in an involuntary manner. For example, Soto and colleagues^4–8^ examined such VWM-based attentional guidance extensively in a line of studies. In their paradigm, participants were required to perform a visual search task during the retaining period of a VWM task. Soto and colleagues found that the item in the visual display matching the features of the memorized item received attentional priority, even when participants knew that this item was always a distractor and hence interfered with the current visual search task. Importantly, memory-driven attentional guidance was a general phenomenon, as it was observed in different situations such as pop-out visual search^4^, or under high cognitive load conditions^5^, and with different measures such as standard reaction times^9^ (RTs) and oculomotor responses (on the first fixation^7^).

When maintaining two items in VWM, however, the evidence is mixed. On the one hand, some studies reported no significant guidance from two simultaneously maintained items. For example, Peters and colleagues^10^ asked participants to simultaneously hold a target and another non-target item in VWM and then to judge whether the target appeared in the following rapid serial visual presentation task. They observed that the distractor matching the features of the memorized items elicited comparable ERP responses to those elicited by distractors matching no features of the memorized items. These results were also supported by other studies (e.g.,^11,12^; but see^13^). On the other hand, other studies reported reliable VWM-based guidance by both items using different experimental paradigms such as the rapid serial visual presentation^14^, manipulations of attentional control settings^15^, and cueing procedures^16,17^.

Some researchers argued that discrepant findings could be attributed to the status of the representations of the items in VWM driven by the task set in the different studies. In this regard, it should be noted that different types of visual search paradigms have been used to investigate this topic. The specific visual search paradigm implemented in a given study can indeed induce the representations of the items held in VWM to assume different priority levels^10,11,18,19^. When the experimental setting requires participants to memorize two items^10–12^, the target template would be necessarily stored in VWM^20,21^ with an active or prioritized status^18^, whereas the other non-target VWM item would become less relevant and would be characterized by an accessory status^10^. Thus, not only would the target template have priority to guide attention, but this would also result in blocking or even suppressing the guidance from the accessory VWM items^10,22^. Since in studies reporting no attentional guidance from the accessory item representation^10–12,19^, one of the items to be held in VWM was specified as target template of the visual search task, it is reasonable to assume that the participants implicitly assigned it an active, high-priority, status, which in turn resulted in weakening the guidance, if any, from the accessory item held in VWM. Recently, in order to clarify this issue, and to constraint the strategy for selective encoding of the items, van Moorselaar *et al*.^23^ have firstly asked participants to keep two items in VWM and did not specify either of them as the search template. Then, in order to manipulate the status of VWM representations, prior to the visual search task and the subsequent memory tests, a cue was displayed to indicate which of the VWM items would be asked to be remembered first. According to van Moorselaar *et al*.^23^, this procedure would induce participants to prioritize the status of the cued item, and to decreasing the priority of the other to-be-remembered item, thus downgrading it to an accessory status. The results showed that the prioritized VWM item was able to guide attention to the VWM-matched singleton distractor in the visual search display, whereas the accessory VWM item was not. Further, van Moorselaar *et al*.^23^ found that without prioritizing one item over the other, none of the items maintained in VWM could bias attention. van Moorselaar *et al*.^23^ argued that when multiple representations are simultaneously held in VWM, only the prioritized representation could guide visual attention (i.e., the “single-item-template hypothesis^18^). In addition, when all VWM items were made equally relevant, both of the items were likely to be assigned an accessory status, which, in turn, could account for the observation that none of them exerted attentional guidance.

Hollingworth and Beck^24^ have recently reexamined the single-item-template hypothesis with two different types of visual search task. One such task was the singleton-shape search task used by van Moorselaar *et al*.^23^ and the other was a gap-location search task that required focal attention to search for the gap location in outline shape stimuli. The results showed significant VWM-based attentional guidance in the gap-location search task but not in the singleton-shape search task when one distractor in the visual search display matched anyone of the two VWM representations (i.e., “Mem-2 Match-1” condition). Hollingworth and Beck^24^ attributed this discrepancy to a possible difference in sensitivity of the two visual search tasks on VWM-based capture. Moreover, they found that when two distractors in the gap-location search task respectively matched the two VWM representations (i.e., “Mem-2 Match-2” condition), the guidance was enhanced relative to the “Mem-2 Match-1” condition. These results are not in line with the single-item-template hypothesis and, in contrast, support the conclusion that multiple representations in VWM can simultaneously exert an influence on visual attention (see also Chen & Feng^25^), which is referred to as the multiple-item-template hypothesis^24^.

In keeping with Hollingworth and Beck^24^, the conclusion that the accessory VWM representation could not guide attention in van Moorselaar *et al*.^23^ may be constrained by the type of visual search task used in that study. The present study was aimed at clarifying this issue and to further test whether, when adopting more sensitive visual search tasks, the accessory VWM representation, which is suppressed by the prioritized VWM representation^22^, can guide attention or not. Whereas Hollingworth and Beck^24^ were primarily interested in comparing high (2 items) and low (1 item) memory load, here we focused on high memory load in a situation in which the two items were likely to receive a different mnestic priority.

In order to investigate the attentional guidance from the accessory VWM items, we implemented a variant of the paradigm used by van Moorselaar *et al*.^23^, with two crucial modifications. Firstly, the singleton-shape search task was replaced with a tilted-line visual search task similar to that used by Soto *et al*.^26^. Since the tilted-line search task was similar to the gap-location search task (i.e., both were likely to require focal attention), this would maximize sensitivity to VWM-based attentional guidance in keeping with Hollingworth and Beck^24^. Critically, the tilted-line search task has been shown to be effective in detecting VWM-based attentional guidance, at least when participants have to memorize just one item^4,5,7,8^. The adoption of a visual search task requiring focal attention in the present study was aimed at favoring a closer comparison with other related studies and, more specifically, at clarifying the possible reason underlying the lack of VWM-based guidance from the accessory item reported by van Moorselaar *et al*.^23^. Secondly, eye movements were tracked during the visual search task. The link between saccadic eye movements and attention is well established^27–29^. Moreover, previous studies have shown that VWM based attentional guidance could occur at early stages during visual search^4,7,30^. For this reason, eye movement dynamics and measures such as the first fixation proportion (FFP) might represent reliable (and ecological) dependent variables better suited to capture VWM-based guidance of attention. In contrast, manual RTs, which are the standard measure in this specific research topic, represent the behavioral output of a series of cognitive processing stages from perception to response execution. Accordingly, unlike many previous studies, in the present set of experiments we decided to capitalize on eye movements as they represent a more direct, online measure of visual attention. In this regards, it is also worth noting that there is evidence according to which the VWM-based attentional guidance effect as measured in RTs can be weakened or even reversed by cognitive control^31–33^. This entails that, compared to FFPs and early oculomotor dynamics, RTs are more prone to cognitive control and therefore more vulnerable and sensitive to strategic factors that might, in principle, affect and mask the phenomenon under investigation^34^.

In sum, the goal of the present study was to investigate VWM-based guidance of attention focusing on a situation in which two items were to be maintained in VWM with a different mnestic priority. The adoption of a visual search task likely requiring focused attention combined with manual but also eye-movement measures should set the most sensitive experimental conditions to detect VWM-based guidance of attention. Based on the multiple-item-template hypothesis^24^, we expected to observe attentional guidance not only by the prioritized item but also by the accessory item, especially on FFPs. The lack of guidance by the accessory item would instead lend support to the “single-item-template hypothesis”^24^.

## Experiment 1

### Method

#### Participants

Sample size was based on previous studies exploring this phenomenon focusing on eye movement measures^12,16^. Sixteen students (7 men, aged between 19 and 24 years old, mean age = 21.5 years) with normal or corrected-to-normal visual acuity and normal color vision took part in this experiment. All were right-handed. An informed consent was obtained at the beginning of the session following a research protocol approved by the Research Ethics Committee of Guangzhou University. All methods used in all experiments performed during the present study were performed in accordance with the relevant guidelines and regulations of the research ethics committee.

#### Apparatus and design

The experiment was conducted in a sound-attenuated and dimly lit room. All visual stimuli were presented with a gray background on a color CRT monitor (resolution: 1024 × 768; frame rate: 100 Hz) about 70 cm away from the participant. Eye movements were recorded from the right eye during the search displays by means of an Eye Link 1000 (1000-Hz temporal resolution) infrared head-mounted eye tracker (SR Research Ltd., Mississauga, Ontario, Canada). At the beginning of each block the participants performed a standard nine-point grid calibration. An adjustable chin rest helped to maintain head position.

#### Stimuli and procedure

The stimuli were drawn by crossing the features of five colors (red, blue, yellow, green, and pink) and five line drawings of shapes (square, triangle, circle, diamond and hexagon). The size of the shapes was approximately 1.9° × 1.9° and the thickness of the border line of the shapes was about 0.5°.

**Figure 1:**
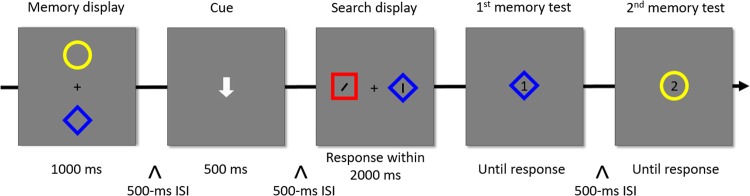
Schematic illustration of the sequence of events in Experiment 1.

The procedure was similar to that employed by van Moorselaar *et al*.^23^. As shown in Fig. 1, our paradigm combined a VWM task with a visual search task. The VWM task included a memory display, a retrieval cue and two memory tests. The memory display consisted of two colored shapes positioned at a distance of 2° above and below of the central fixation cross (0.5° × 0.5°). The retrieval cue was a white up- or down-arrow presented in the center of screen. The memorized item located at the position indicated by the arrow was probed by the 1^st^ memory test, and the other was probed by the 2^nd^ memory test. In each memory test, an item appeared at the center of the screen and the participants were asked to judge whether it was the same as the memorized item or not by pressing the “F” and “J” keys of a standard keyboard. “yes” and “no” response trials occurred with the same probability in an unpredictable manner. In the “no” response trials, the item in memory test differed from the memorized item in color, shape, or both features equiprobably.

The visual search display consisted of a target item and a distractor item randomly positioned 6° left and right of a central fixation cross (0.5° × 0.5°). The target item was a colored shape containing a tilted line (38° either to left or to right), and the distractor item was another colored shape containing a vertical line. Both lines were black and with the size of 0.57° × 1.2°. The target and the distractor had no overlapping features. The participants were required to judge the orientation of the tilted line by pressing the “F” key for left-tilted segments and the “J” key for right-tilted segments.

Each trial began with a small white dot (radius = 0.5°) in the center of the screen, which was used for drift correction. Once the eyes of the participants were detected as fixating on the dot for more than 1000 ms, the experimenter immediately pressed the space bar to trigger the appearance of the memory display, which lasted 1000 ms. After a 500-ms Inter Stimulus Interval (ISI), during which only the fixation point was visible, a retrieval cue was presented for 500 ms. After another 500-ms ISI frame, the visual search display appeared, and the participants were required to respond to the target as quickly and accurately as possible. Participants were given a maximum of 2000 ms to respond. As soon as a response was provided or the deadline had expired, the 1^st^ and the 2^nd^ memory test were successively displayed with a separation of 500-ms ISI frame. The participants were instructed to respond accurately to each memory test without time pressure. After the response to the 2^nd^ memory test, a display in the screen was shown to indicate to the participants that they should press the space bar to initiate the next trial.

The distractor in the visual search display might match both color and shape of either the cued memorized item in the “match 1^st^” condition, or the uncued memorized item in the “match 2^nd^” condition, or did not match any features of the memorized items in the control condition. Each matching type included 72 trials, evenly intermixed within 3 experimental blocks. Before the experimental blocks, each participant had to complete a 20 trial practice phase to get familiar with the experimental task.

### Results

#### Accuracy

The mean accuracy for the 1^st^ memory test (M = 89.31%, SD = 3.91) was significantly higher than the 2^nd^ memory test (M = 83.94%, SD = 5.81), *t*(15) = 5.17, *p* = 0.0005). The mean accuracy for the search task was reasonably good (M = 98.18%, SD = 0.96) and not analyzed further. The analyses for manual RTs and eye movement data were conducted including only trials with correct responses in both the search task and the memory test task.

#### Search RTs

**Figure 2:**
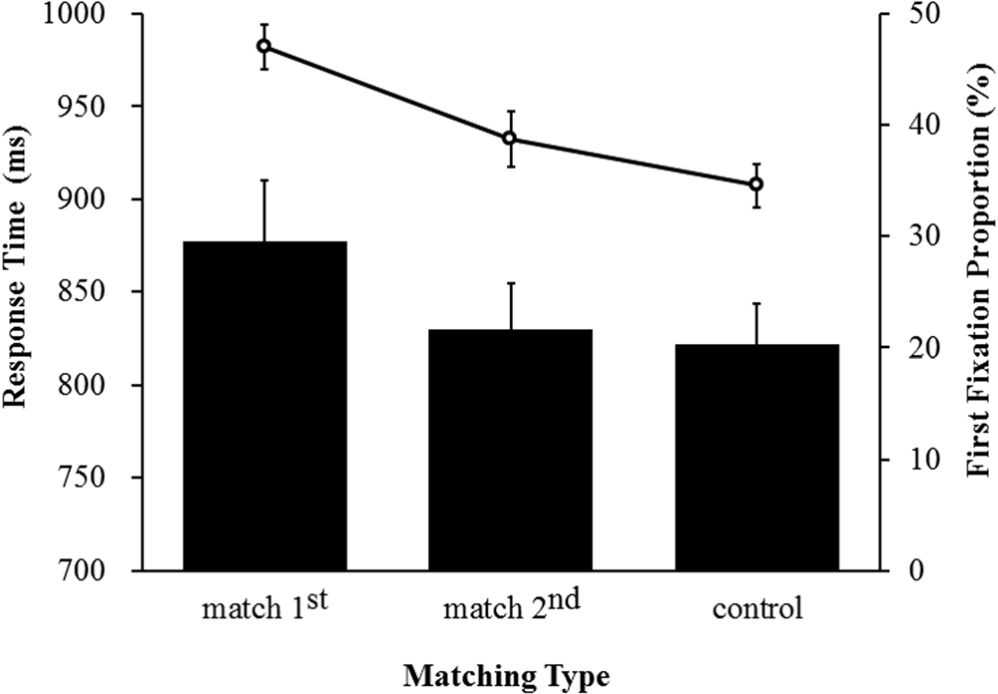
Mean RTs and FFPs as a function of matching type in Experiment 1. The bar plot and the line plot represent RTs and FFPs respectively. Error bars are standard errors.

RTs exceeding 3 SDs of each participant’s mean RT were discarded as outliers. The data were then submitted to a repeated-measures ANOVA with matching type as factor. The analysis showed a significant main effect, *F*(2,30) = 7.35, *p* = 0.0025, η^2^_p_ = 0.33. Two-tailed *t*-tests revealed that the mean RTs for the “match 1^st^” trials (M = 877 ms, SD = 132) were significantly greater than RTs for both the “match 2^nd^” trials (M = 830 ms, SD = 100, *t*(15) = 3.25, *p* = 0.005) and the control trials (M = 822 ms, SD = 90, *t*(15) = 2.79, *p* = 0.014), whereas the difference between the two latter conditions was not significant (*t*(15) = 0.72, *p* = 0.490) (see Fig. 2). These results are consistent with those reported in previous studies^10–12,23^, suggesting that the prioritized VWM representation can guide visual attention whereas the accessory VWM representation cannot.

#### FFPs in search task

We further estimated the VWM based attentional guidance on FFPs that fell in the region of three matching types of distractors (i.e., a virtual circle region with a radius of 2° around the distractor) in the visual search task. FPPs were limited to the first saccadic eye movement time-locked to the onset of the visual search array. In computing the FFPs, we discarded the trials in which (1) the actual first fixations were not located in the region of central fixation (2° around the central fixation); (2) the first eye movements were less than 100 ms and greater than 500 ms. The repeated-measures ANOVA highlighted a significant main effect of matching type, *F*(2,30) = 17.54, *p* = 0.0001, η^2^_p_ = 0.54. Multiple comparisons further revealed that the FFP (see Fig. 2) in the “match 1^st^” condition (M = 47.02%, SD = 8.22%) was significantly higher than in both the “match 2^nd^” condition (M = 38.77%; SD = 9.92%, *t*(15) = 3.82, *p* = 0.002) and the control condition (M = 34.57%; SD = 7.86%, *t*(15) = 5.36, *p* = 0.001). However, in sharp contrast with RT results, FFP in the “match 2^nd^” condition was also significantly higher than in the control condition (*t*(15) = 2.19, *p* = 0.045), suggesting the accessory VWM representation was able to guide attention, although the magnitude of this effect was apparently smaller than that of the prioritized VWM representation.

## Experiment 2

Experiment 1 adopted an experimental procedure similar to that employed by van Moorselaar *et al*.^23^ and replicated their RT results, but revealed a different pattern as concerns FFPs. As described earlier, FFPs and RTs have very different time courses, and FFPs (and oculomotor dynamics in general) are better suited to reveal the effects on early stages of visual search. Previous researchers have already reported diverging results when comparing FFPs and manual RTs^34,35^. The present findings seem to confirm that FFPs are more sensitive to detect the occurrence of attentional guidance as compared to manual RTs.

**Figure 3:**
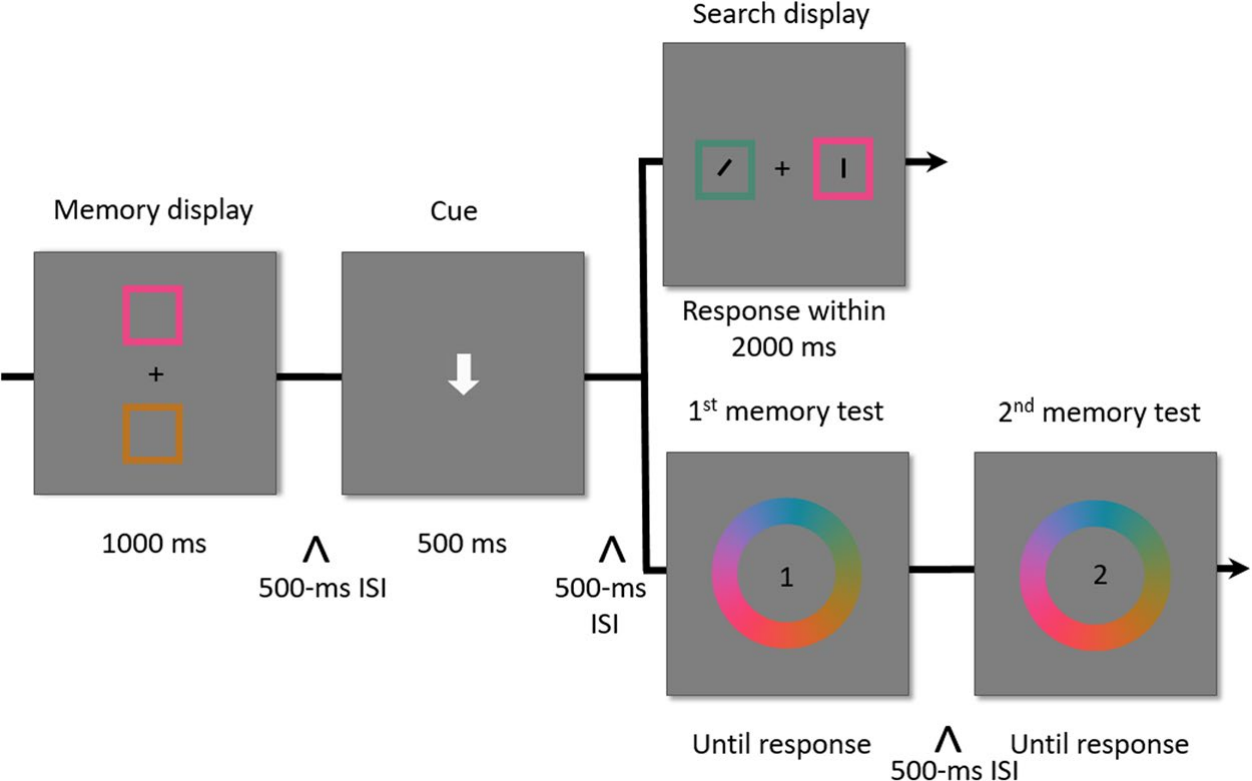
Schematic illustration of the sequence of events in Experiment 2.

In both the Experiment 4 of van Moorselaar *et al*.^23^ and Experiment 1 of the present study, the retrieval cue was always followed by the visual search task with a fixed temporal interval. This, in turn, might have lead the participants to intentionally prepare for the visual search task before the appearance of the search display. It might be argued that these temporal dynamics could have affected performance in the visual search task. In Experiment 2, we aimed to address this issue by preventing participants to predict the temporal occurrence of the visual search task. This was achieved by presenting either the visual search task or the memory tests after cue offset, in an unpredictable manner (see Fig. 3). In addition, it should be noted that the to-be-memorized items in Experiment 1 were easy to verbalize, and van Moorselaar *et al*.^23^ argued that the verbal coding of the accessory VWM item might play a role in affecting attentional guidance by VWM items. In order to decrease the possibility of verbalizing the memorized items, in Experiment 2, we replaced the memory test used in Experiment 1 with a color wheel memory test which was also used in previous studies^23,36^.

In sum, Experiment 2 was aimed to implement a more robust experimental design with respect to the previous experiment, and to provide additional evidence concerning attentional guidance from the accessory VWM representation. The predictions were the same as in the previous experiment. Based on the results of Experiment 1, we expected to observe attentional guidance not only by the prioritized item but also from by the accessory WM representation item, especially on FFPs, in line with the multiple-item-template hypothesis^24^. The lack of guidance would instead lend support to the possibility that the results observed in Experiment 1 were the consequence of strategic factors such as verbal recoding or temporal expectancy.

### Method

#### Participants

Another group of sixteen students (8 men, aged between 18 and 23 years old, mean age = 20.8 years) participated in Experiment 2. None of them had taken part in Experiment 1. An informed consent was obtained at the beginning of the session following a research protocol approved by the Research Ethics Committee of Guangzhou University.

#### Apparatus and design

As shown in Fig. 3, the experimental procedure was similar to that used in Experiment 1, with two exceptions. First, all stimuli were squares, and the colors of all memorized and search items in each trial were randomly selected from 180 colors of the color wheel with the restriction of separating each other at least 72°, as in previous studies^23,37^. Second, after the retrieval cue and the 500-ms ISI, the participants were presented with either only the visual search task, or only the two successive memory tests. The visual search task and the memory tests occurred with the same frequency and were administered in an unpredictable fashion. As concerns the memory tests, the participants clicked on a standard color wheel^38^ to report the identity of the color of the memorized items.

### Results

#### Accuracy

The mean accuracy for the search task was 98.47% (SD = 1.8). Considering that the precision of VWM representations has little impact on attentional guidance^23,36^, only the average deviation error along the color wheel of the memory tests were computed. These indicated that the memory deviation was greater for the 2^nd^ memory test (M = 15.53°, SD = 3.01) than the 1^st^ memory test (M = 11.40°, SD = 3.22), *t*(15) = 9.21, *p* = 0.0001.

#### Search RTs

**Figure 4:**
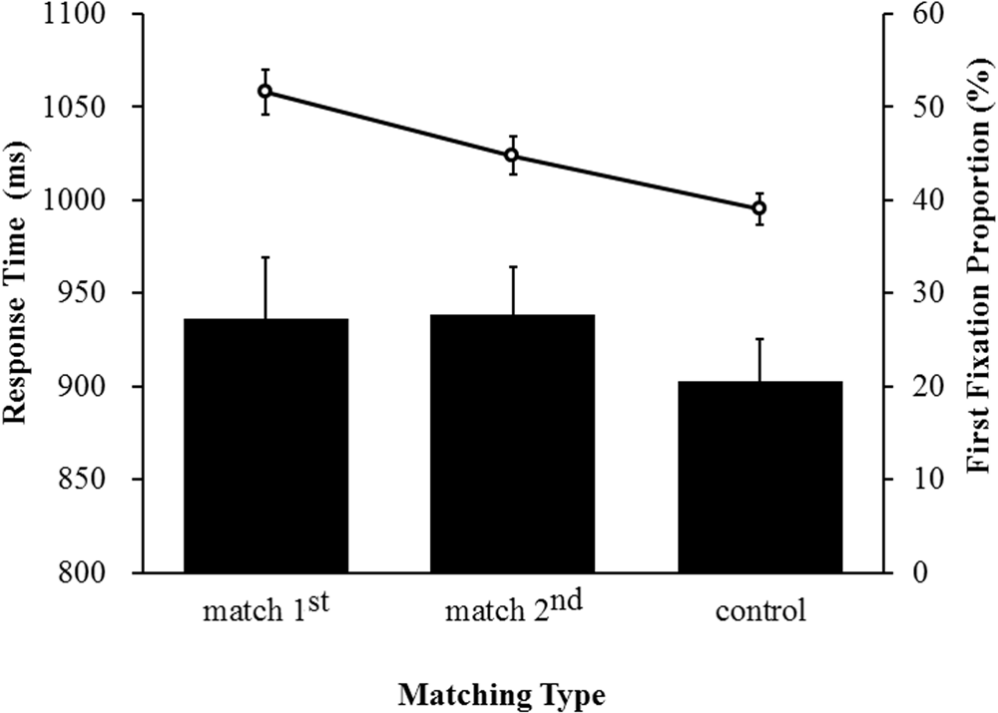
Mean RTs and FFPs as a function of matching type in Experiment 2. The bar plot and the line plot represent RTs and FFPs respectively. Error bars are standard errors.

RT data were handled in the same manner as in the previous experiment. The results of the repeated-measures ANOVA for search RTs showed a significant main effect of matching type, *F*(2,30) = 3.67, *p* = 0.039, η^2^_p_ = 0.21. Two-tailed *t*-tests showed that mean RTs (see Fig. 4) for the “match 1^st^” (M = 936 ms, SD = 197) and “match 2^nd^” condition (M = 939 ms, SD = 164) were nearly identical (*t*(15) = 0.19, *p* = 0.85), but both were significantly greater than RTs in the control condition (M = 903 ms, SD = 159, *t*s(15) > 2.23, *p*s < 0.05). This pattern suggests that regardless of whether sharing common features with either the prioritized or the accessory VWM representation, the distractor captured attention to the same extent.

#### Search FFP

FFP detection was implemented using the same constraints as in the previous experiment. The results of the ANOVA for FFPs showed a significant main effect of matching type, *F*(2,30) = 10.62, *p* = 0.0004, η^2^_p_ = 0.43. As shown in Fig. 4, two-tailed t-tests showed that FFPs in the “match 2^nd^” condition (M = 44.79%, SD = 7.80) were significantly greater than in the control condition (M = 39.02%, SD = 6.25, *t*(15) = 3.08, *p* = 0.008), but significantly smaller than in the “match 1^st^” condition (M = 51.56, SD = 10.32, *t*(15) = 2.41, *p* = 0.031). These results replicated the findings of Experiment 1, indicating that the accessory VWM representation could indeed guide attention although the magnitude of the guidance was smaller than that exerted by the prioritized VWM representation.

## Discussion

When prioritizing the status of one VWM representation by using a retrieval cue, can the other accessory representations in VWM guide visual attention? The present study addressed this issue by adopting an experimental procedure similar to that used by van Moorselaar *et al*.^23^ with two relevant modifications. Firstly, we changed the visual search task to enhance sensitivity to detect the VWM-based attentional guidance. Secondly, we tracked eye movements in order to provide a more ecological and sensitive estimate of the attentional guidance effect at early stages of visual search. The FFP results of both Experiment 1 and 2 showed that the accessory VWM representation could indeed guide visual attention, although the magnitude of the guidance effect was smaller than that observed for the prioritized VWM representation. The RT results in Experiment 1 replicated the pattern reported by van Moorselaar *et al*.^23^, i.e., no accessory VWM-based guidance was observed. However, when participants were prevented from predicting the appearance of the visual search display and the possibility of verbalizing the memory items was minimized, in Experiment 2, the accessory VWM representation was found to be able to guide attention in the same manner as the prioritized VWM representation also in RT data. Critics may object that not knowing whether the search or the memory test will appear (in Experiment 2) could not only prevent temporal expectancies but also influence memory processes for the two items. Although the different memory measurements of Experiment 1 and Experiment 2 prevent an accurate comparison between the two memory accuracies, it is worth noting that in both experiments the accuracy for the second memorized item was always lower compared to the accuracy for the first item. The observation of a reliable, consistent pattern across the two experiments suggests that the memory processes involved in the two experiments were not strongly dissimilar and this factor did not play a key role in shaping the visual search results.

It could be argued that because in both Experiment 1 and 2 the search array consisted of only two items, this could have led our participants to adopt a spatial recoding-like strategy in that, when a memory-matching distractor was present, this was always counterpredictive with respect to the target location. If this were the case, then the non-matching control condition would be difficult to compare to the two matching conditions. Although we acknowledge this possibility, the overall visual search results of both Experiment 1 and 2 are not consistent with the occurrence of such strategy in our studies. More specifically, had our participants adopted the spatial recoding-like strategy, this should have resulted in a better visual search performance for the match 1^st^ (and possibly also the match 2^nd^) condition as compared to the control condition (in which no matching features could “push” attention to the opposite location). Analysis of the results in both experiments clearly indicates that visual search performance was always better in the control condition, which, in turn, makes the occurrence of a spatial recoding-like strategy unlikely. According to the VWM-state-hypothesis proposed by Olivers *et al*.^18^, the status of VWM representations will affect their interaction with attention, in that the guidance from the accessory representation should be blocked by the prioritized representation in VWM (see also^10,12,23^). The present overall results support this view and confirm the impact of the status of VWM representations on attention guidance, in that the prioritized VWM representation had a greater effectiveness in guiding attention as compared to the accessory VWM representations. However, at variance with the predictions of the VWM-state-hypothesis, the present results clearly suggest that the accessory VWM representation can also bias attention, at least to some extent.

One possibility to account for the discrepancy between the present findings and those reported by van Moorselaar *et al*.^23^ might refer to the different type of visual search task adopted in the two studies. In the additional singleton task of van Moorselaar *et al*.^23^, the attentional guidance is determined on whether the VWM-matched singleton distractor could additionally capture attention. The search mode for this type of task is regarded as the singleton detection mode, and the attentional capture mostly depends on the salience of singleton in search display^39,40^. Obviously, the salience of VWM representations is decreased with increasing the number of VWM representation, and this in turn might possibly determine the absence of attentional guidance from the accessory VWM-related singleton in van Moorselaar *et al*.^21^ and Olivers *et al*.^30^. By contrast, the tilted-line visual search task used in the present study is more likely carried out in a feature-search mode^39^, as it requires focal attention to search for the target rather than monitoring the emergence of the singleton feature. As a result, such search mode would be less susceptible to the salience of the singleton and even override the attentional capture from the singleton itself^41^. Following this reasoning, it could be argued that the feature-search mode task is more sensitive for detecting VWM-based attentional guidance as compared to a singleton detection mode task especially when multiple representations ought to be simultaneously maintained in VWM (see also in Hollingworth and Beck^24^). That said, some researchers^11–13^ adopted a visual search task which can be assumed to engage a feature-search mode, and yet did not find evidence for attentional guidance from accessory VWM representations. The possible reason for this discrepancy might be that the complex shapes used in those studies lack the stimulus attributes that can effectively guide attention^7,13^ or, alternatively, the accessory VWM representation would lose its competitiveness for guiding attention in the face of the stronger competitor, such as the target template^19^.

Interestingly, Hollingworth and Hwang^36^ have used a different manipulation to induce differences in the status of the to-be-memorized items. After the memory array, their participants were presented with a spatial cue that indicated with an 80% validity the item that will be probed in the memory test. This allowed to distinguish cued and uncued items which, following their rationale, may be interpreted as high-status and low-status items respectively. Overall, no evidence for VWM-based guidance emerged for the uncued items^36^. It is not easy to directly compare the present results with those of Hollingworth and Hwang^36^ because in that study visual search performance for the cued items was not assessed. In addition, it might be argued that manipulating expectancies regarding the probed to-be-remembered item might call into play different processes as compared to those involved in the procedure implemented in our studies.

One further important possibility is that the inconsistency between the present results and those reported by both Hollingworth and Hwang^36^ and van Moorselaar *et al*.^23^ may result from the different stages of attentional guidance addressed in the measures collected in the different studies. As anticipated earlier, with respect to manual RTs, FFPs can capture attentional guidance at early stages of visual search and are more impervious to cognitive control. Consistent with this interpretation, the present findings show that when considering the different temporal predictability of the visual search display in Experiment 1 and in Experiment 2, the accessory VWM-based attentional guidance is comparatively stable across experiments when assessed by FFPs, but is prone to variation when assessed with manual RTs. Peters *et al*.^22^ explored the neural activity of attentional guidance from the target template and accessory representations in VWM, and found that the activity in extrastriate visual cortex was enhanced for target-matching visual input, but was suppressed for accessory-matching visual input, thus suggesting that cognitive control would suppress the interference from irrelevant VWM information to promote the focus on task-relevant information. This interpretation, however, cannot rule out the possibility that attention can still be captured by the accessory-matching visual stimulus before its representation is suppressed. Following this logic, it is possible that the null effect reported on manual RTs in van Moorselaar *et al*.^23^ may also be the result of the suppression of the early attentional guidance testified by FFPs in the present experiments.

The single-item-template hypothesis of Olivers *et al*.^18^ suggested that only the attentional template in VWM could guide attention, while the other accessory VWM representations would be downgraded to a passive status by the privileged attentional template, and hence could not bias attention. However, Hollingworth and Beck^24^ and two recent studies by Chen and Feng^25^ and Bahle, Beck and Hollingworth^42^ using different types of experimental manipulations and dependent measures have shown that multiple representations in VWM could guide visual attention simultaneously, thus supporting the multiple-item-template hypothesis. Consistent with these findings, the current results also cast evidence for a robust accessory VWM-based attentional guidance on FFPs. These findings suggest that the guidance from accessory VWM representations cannot be completely overridden by the prioritized VWM representation. Additionally, the current results also indicate that the status of the accessory VWM representation is unlikely to be completely downgraded to a passive status, but remains in active status, at least to to some extent.

In conclusion, the present study clearly indicated that multiple VWM representations, irrespective of whether held in either prioritized status or in accessory status, can guide visual attention. In addition, the strength of attentional guidance was greater for the prioritized representation than for the accessory representations. This latter evidence was observed in both experiments and future studies will need to address whether this pattern can be observed across other types of experimental manipulations and settings, in order to provide indications as to whether VWM-based guidance of attention could go beyond a simple all-or-none dichotomy and reflect a more nuanced prioritization hierarchy.
